# A signature of six-hypoxia-related genes to evaluate the tumor immune microenvironment and predict prognosis in gastric cancer

**DOI:** 10.1186/s12920-022-01411-9

**Published:** 2022-12-16

**Authors:** Kena Zhou, Congbo Cai, Guanjun Ding, Yi He, Di Hu

**Affiliations:** 1 Gastroenterology Department of Ningbo No. 9 Hospital, No.68 Xiangbei Road, Jiangbei District, Ningbo, 315000 Zhejiang China; 2 Emergency Department of Yinzhou No.2 Hospital, Ningbo, 315000 Zhejiang China; 3 General Surgery Department of Ningbo No. 9 Hospital, Ningbo, 315000 Zhejiang China

**Keywords:** Hypoxia, Gastric cancer, TME, Risk model, Immune response

## Abstract

**Background:**

Hypoxia will trigger a series of immunosuppressive process in tumor microenvironment, leading to the progression in gastric cancer (GC). This research aims to establish a prognostic model made up of hypoxia-risk-related genes in GC.

**Methods:**

Hypoxic genes were outlined via the protein–protein interaction network. And a prognostic model was developed using univariate cox analysis and lasso regression from data in TCGA. Two independent queues of GEO were used for validation.

**Results:**

We set up a hypoxic model presented as an independent prognostic factor for GC. And a nomogram combined this model with clinical features can predict OS with great performance. Furthermore, DNA methylation, IHC and cell line analyses validated the expression of hypoxic genes in GC.

**Conclusions:**

In summary, we proposed and verified a hypoxia-risk-related model, which could reflect the immune microenvironment and predict prognosis in GC.

**Supplementary Information:**

The online version contains supplementary material available at 10.1186/s12920-022-01411-9.

## Introduction

Gastric cancer (GC) is one of the most common malignant tumors in digestive tract, ranking fifth in cancer incidence and third in cancer mortality worldwide [1]. The coronavirus disease 2019 (COVID-19) pandemic has a significant impact on early diagnosis and treatment of GC, leading to an obvious increase on the mortality rate in cancer [2]. It is clear that treatment effect on advanced GC is quite poor. And evaluation of overall survival (OS) in GC patients is emphasized on the choice of clinical treatment options.

GC is a highly heterogeneous tumor, which is not only affected by its own genetic factors, but also has a complex relationship with the tumor microenvironment (TME) [3]. Hypoxia is an important manifestation of the TME. Due to insufficient oxygen and nutrient supply, tumors are likely to grow up rapidly [4, 5]. Emerging studies have shown that hypoxia could induce tumor malignancy, including progression, invasion and metastasis [6, 7]. Besides, hypoxia drives tumor immunosuppression and immune escape [8, 9]. Previous evidence indicated that hypoxia in the microenvironment of GC could predict the prognosis of patients via direct or indirect way [10]. Therefore, we hypothesize that hypoxic genes could be good predictions and reflect TME in GC. Currently, there is no hypoxia-related gene signature for GC.

In this paper, we briefly introduce a prognostic model composed of hypoxia-related genes. This model can evaluate OS of GC patients, as well as provide potential view of using hypoxic modulators in targeted therapy and immunotherapy.

## Materials and methods

### Datasets

The experimental set was obtained from the Cancer Genome Atlas (TCGA) database (https://portal.gdc.cancer.gov, November 5, 2020) consisted of 321 patients from “TCGA-STAD”. STAD batch RNA-seq data, gene mutation information, and clinicopathological features were downloaded from the TCGA database. And the verification set was downloaded from the Gene Expression Omnibus (GEO) database (https://www.ncbi.nlm.nih.gov/geo/, November 12, 2020) composed of 433 patients (GSE84437) and 192 patients (GSE15459) altogether. Both the TCGA and GEO databases are publicly available worldwide. Thus there’s no need for institutional ethics committee approval or patients’ informed consent. Every clinical data was checked, and patients with missing clinical information or lack of complete follow-up information were excluded in the univariate and multivariate analyses process.

### Establishment of a risk model

Firstly, hypoxia-related genes were downloaded from the GSEA website (https://www.gsea-msigdb.org/). Secondly, a protein–protein interaction network (PPI) was generated with the application of STRING (https://www.string-db.org/). And the top 50 most relevant hypoxic genes were selected out for further study. Univariate cox analysis was used for the 50 hypoxic genes, and genes with *P* < 0.05 were secondarily screened out for later research. A compatible model was built up by LASSO regression because this method could retain valuable variables and avoid overfitting [11]. And the risk-score formula was calculated as: ${\text{Risk score}}={\sum }_{\mathfrak{i}=1}^{\mathrm{N}}(\mathrm{Ei}*\mathrm{Ci})$; while N = 6, Ei was the expression value of each hypoxic gene, and the Ci was the corresponding multivariable Cox regression coefficient. The Principal Component Analysis (PCA) method was used to evaluate the reliability of the model, and PCA was implemented with the R package “Rtsne”.

### Validation for clinical value

Risk scores of all patients in TCGA were calculated on the basis of the prognosis model. And subjects were divided into high- and low-risk groups according to the intermediate risk value. Same risk score criteria was utilized to divide verification set into high- and low-risk groups. Survival curves were drawn in R language to make comparison between the two groups using the log-rank test. Likewise, 1–3–5-year ROC curves were also produced in R language to verify the accuracy of the risk model in predicting OS. Univariate and multivariate Cox regression analysis were used to determine whether the prognostic model was an independent factor or not. Concordance index (C-index) of the model was calculated using the R package "survcomp", which further illustrated the predictive ability of the model. The R package “rms” was used to establish a nomogram for all risk factors for the OS in GC patients.

### Analysis on infiltrating of immune factors

We used three methods to explore immune cells infiltration. Firstly, we used the CIBERSORT algorithm [12] to assess the relative abundance of tumor infiltrating immune cells in different risk groups. This is a new calculation method developed by Newman et al. [13], which can evaluate the proportion of 22 immune cells in all recruited patients from the TCGA and GEO, including T cells, B cells, neutrophils, macrophages, natural killer cells, dendritic cells, plasma cells and eosinophils. Secondly, the enrichment levels of 29 immune-related functions between the two groups were assessed by single-sample gene set enrichment analysis (ssGSEA) scores. Thirdly, we also performed immune infiltration analysis using the TIMER (https://cistrome.shinyapps.io/timer/) platform. Possible response to immunotherapy was evaluated through Tumor Immune Dysfunction and Exclusion (TIDE) algorithm (https://tide.dfci.harvard.edu).

### DNA methylation, gene mutation analysis and expression validation

A web tool named MethSurv (https://biit.cs.ut.ee/methsurv/) was used to assess the prognostic value of different CpG methylation patterns of the six hypoxic genes in GC patients. In addition, two waterfall plots were made to illustrate the detailed gene mutation characteristics between high- and low-risk groups via “oncoplot” function in R software using “maftools” package. Human Protein Atlas Database (https://www.proteinatlas.org/) was searched to verify the protein expression level of hypoxic genes. Cancer Cell Line Encyclopedia Database (https://sites.broadinstitute.org/ccle) was applied to validate the expression level of hypoxic gene in the Gastric Adenocarcinoma cell line. Here in this paper, we used the original data of version No. Public 22Q2.

### Statistical analysis

R software (version 4.1.1) and GSEA (version 4.1.0) were used to perform statistical and pathway analysis during the current research. The t-test was used to compare numerical variables. The categorical variables were compared using the chi-squared test. The relative differences among three or more groups were analyzed by one-way ANOVA. The Kaplan–Meier analysis was used to explore the survival difference between high- and low-hypoxia risk groups. A *P* value < 0.05 of a two-tail should be present to be significant.

## Results

### Establishment of the hypoxia-risk-related prognostic model

The general clinical information of the study from TCGA and GEO are detailed in Table 1.

**Table 1:** Summary of clinical characteristic of GC patients

Characteristics	Training group (TCGA, N = 380)	Test group (GSE84437, N = 433)	Test group (GSE15459, N = 192)
Age category			
< 65/≥ 65/NA	156/220/4	267/166/0	83/109/0
Gender			
Male/female	243/137	296/137	125/67
Vital status			
Alive/dead	231/149	224/209	97/95
Grade			
G1/G2/G3/GX	10/140/221/9	NA	NA
Tumor stage			
I/II/III/IV/NA	55/112/151/39/23	NA	31/29/72/60/0
T stage			
T1/T2/T3/T4/NA	20/84/169/99/8	11/38/92/292/0	NA
M stage			
M0/M1/MX	334/26/20	NA	NA
N stage			
N0/N1/N2/N3/NX	113/99/75/75/18	80/188/132/33/0	NA

NA: Clinical data are unknown

**Fig. 1:**
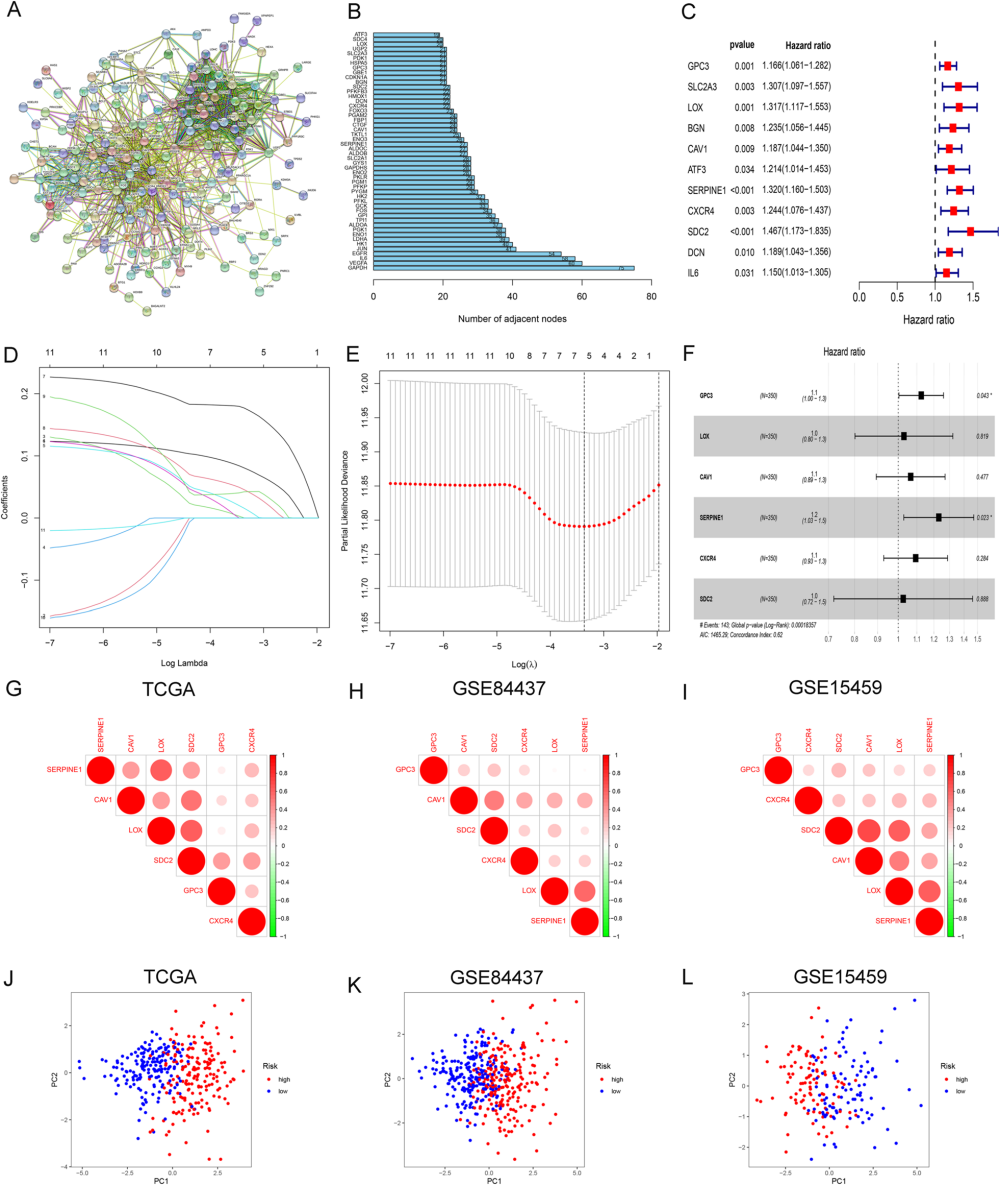
Characterization of hypoxic risk signature to predict prognosis in GC. **A** Protein–Protein Interaction network of 200 hypoxia-relevant genes. **B** Top 50 genes coping with hypoxia. **C** 11 hypoxia associated genes closely related to OS in GC by univariate Cox regression. **D** and **E** Model establishment via LASSO regression. **F** Construction of a hypoxia risk signature to predict prognosis in GC. **G**–**I** Spearman correlation analysis of six hypoxic genes in the TCGA and GEO (GSE84437, GSE15459) databases. **J**–**L** Principal Component Analysis (PCA) analysis between the two risk groups based on hypoxic genes (TCGA and GEO)

Genes related to hypoxia can be downloaded from "h.all.v7.2.symbols", including 200 genes upregulated due to low oxygen level (See S1 for details). Furthermore, we used the STRING database (https://string-db.org/) to construct a PPI network for these 200 hypoxia-related genes (Fig. 1A). The top 50 genes were identified, closely associated with hypoxia (Fig. 1B). In order to explore the prognosis value of hypoxic genes for OS in GC patients, we performed univariate Cox regression analysis on the above 50 genes in the TCGA. The result indicated that 11 hypoxic genes were closely related to the OS in GC patients (*P* < 0.05) (Fig. 1C). And a prognostic model of six hypoxic genes was set up according to the lamda value after LASSO regression (Fig. 1D–F). Our observations in TCGA suggested that there was obvious correlation among all six genes (Fig. 1G). And the similarly significant correlation was also found in the GEO validation groups (Fig. 1H, I).The results demonstrated that the two groups could be distinguished and displayed different hypoxic status according to the hypoxic prognostic model (Fig. 1J–L).

### Prognostic value of hypoxic risk in GC

**Fig. 2:**
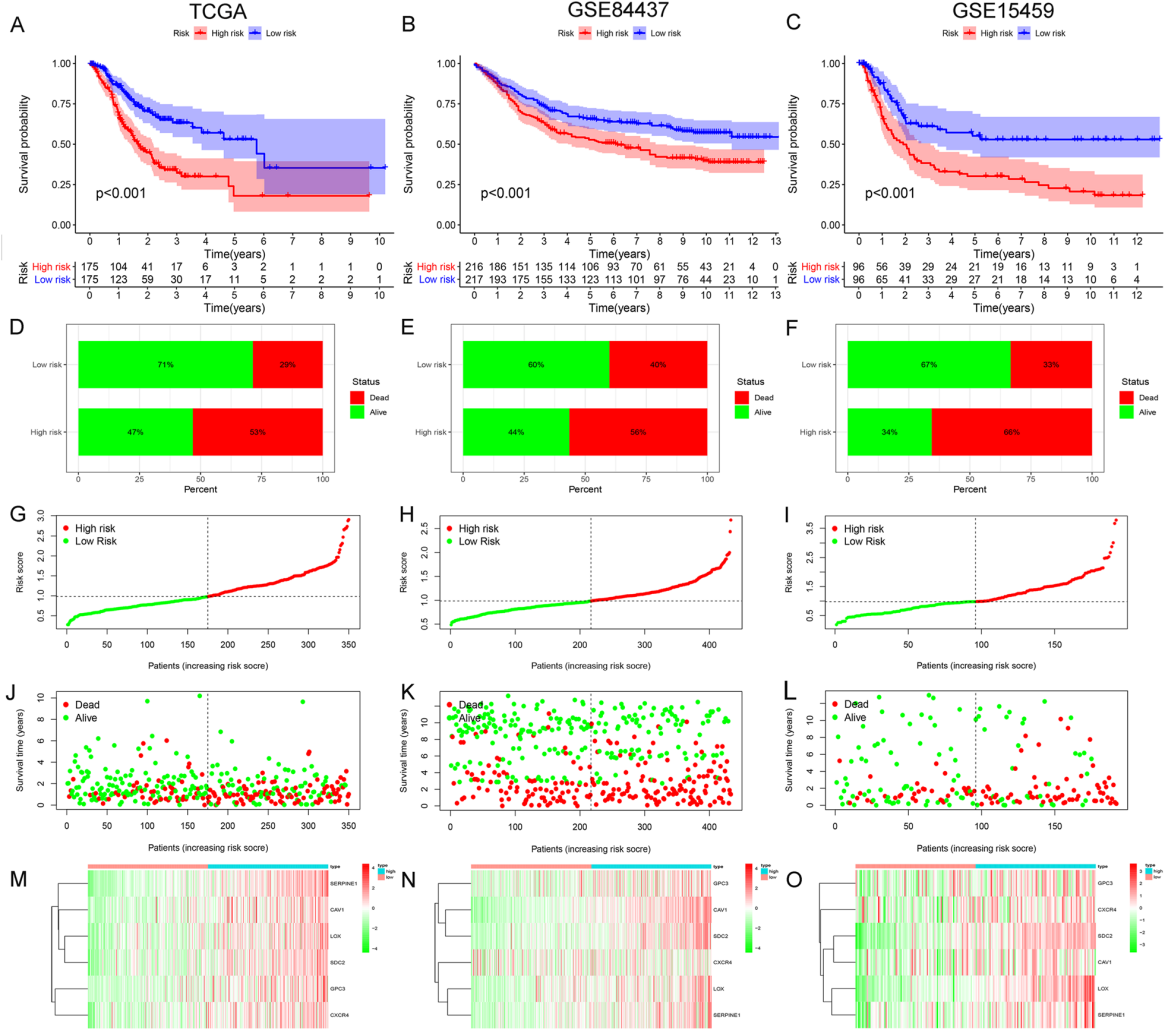
Prognostic value of hypoxic risk in GC. **A**–**C** Kaplan–Meier curves for patients of high- and low-hypoxic risk groups in TCGA and GEO (GSE84437, GSE15459) databases. **D**–**F** Mortality rates of the high- and low-hypoxic risk groups in TCGA and GEO (GSE84437, GSE15459) databases. **G**–**I** Risk values are sorted and the dotted line in the middle divides patients into high- and low-risk groups (TCGA and GEO databases). The green dots are low-risk patients, and the red dots are high-risk patients. **J**–**L** Patient status distribution in the high- and low-hypoxic risk groups. The dot presents patient status ranked by the increasing risk score, where death is in red and alive is in green. The X axis is patient number and Y axis is survival time. **M**–**O** Heat maps showing six hypoxic gene expression profiles in high- and low-hypoxic risk groups from the TCGA and GEO databases

Since hypoxia usually promotes a more aggressive tumor phenotype, we investigated the prognostic value of the hypoxic risk signature. The K–M curve showed that the survival rate of patients in the high-risk group from TCGA was significantly decreased (*P* < 0.01) (Fig. 2A). And it was correspondingly verified in GEO groups (*P* < 0.01) (Fig. 2B, C). Our data also demonstrated that the mortality rate in the high-risk group was higher than that in the low-risk group (Fig. 2D). And the same performance was also observed in GEO for verification (Fig. 2E, F). Sorting all data of TCGA patients by sequencing the risk score, patients were divided into high- and low-risk groups according to the median value (Fig. 2G). As the risk value increases, survival time decreases, and mortality increases (Fig. 2J). And the expression of the six hypoxic genes increased while the risk value increased (Fig. 2M). The risk curves of the independent verification group GSE84437 (Fig. 2H, K, N) and GSE15459 (Fig. 2I, L, O) can further verify the prognostic value of the model.

### Evaluation of the predictive ability of the hypoxic model

**Fig. 3:**
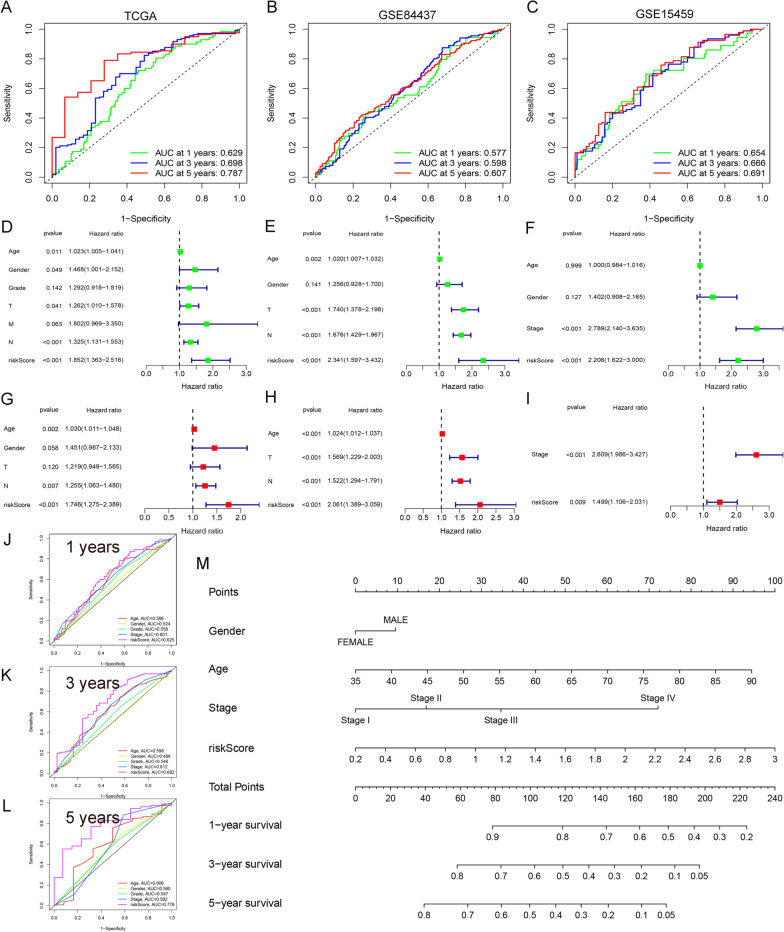
Evaluation of the ability of hypoxic prognostic model. **A**–**C** ROC curves showing the predictive efficiency of the hypoxia risk model of 1-, 3-, and 5-year survival rate. **D**–**F** Univariate Cox analyses evaluating the independent prognostic value of the hypoxic signature in terms of OS in GC. **G**–**I** Multivariate Cox analyses evaluating the independent prognostic value of the hypoxic signature in terms of OS in GC. **J**–**L** ROC analysis of various clinical signatures and hypoxic gene model for 1–3–5 years OS in GC patients from TCGA. **M** The nomogram consisted scores of the six-hypoxic gene model, Gender, age, and stage

To further investigate the predictive value of the established prognostic model in 1-, 3- and 5-year survival rates, we performed the receiver operating characteristic (ROC) curve of the patients from TCGA. The AUC value of the TGCA cohort was 0.629 at 1 year, 0.698 at 3 years, and 0.787 at 5 years (Fig. 3A). This result was further validated in the GEO (Fig. 3B, C). The C-index value of the prognostic model in the TCGA experimental group was 0.6203 (95% CI 57.4–66.6%, *P* < 2.6471E−7). The C-index value in the GSE84437 validation group was 0.5848 (95% CI 54.7–62.2.0%, *P* < 1.1617E−5). The C-index value in the GSE15459 validation group was 0.6265 (95% CI 56.6–68.7%, *P* < 4.6111E−5). The *P* values of the established model in both the experimental group and the validation group were all < 0.01, indicating that the model had strong predictive ability. Moreover, the predictive value of the established signature was confirmed compared with other clinical characteristics (Fig. 3J–L).

Univariate and multivariate Cox analyses were used to evaluate the independent prognostic value of the proposed model for OS in GC patients. Univariate analysis showed that a high hypoxia-risk score was especially related to OS rather than other variables such as age, gender, T and N stages (Fig. 3D). Multivariate analysis showed that the hypoxia-risk score was significantly correlated with the OS in GC patients (Fig. 3G). Consequently, this model could be regarded as an independent prognostic factor in predicting OS in GC. Two external independent cohorts of GSE84437 (Fig. 3E, H) and GSE15459 (Fig. 3F, I) verified this result. Furthermore, drawing a vertical line in the nomogram allows the calculation of individual risk scores composed of prognostic model value and traditional clinical data. Thus, accurate 1-, 3-, and 5-year OS in GC patients can be obtained directly (Fig. 3M).

### Immune status of the hypoxia-risk prognosis model

A considerable amount of research suggest that the hypoxic microenvironment can protect tumors from natural anti-tumor immune responses by inhibiting anti-tumor immune effector cells and promoting immune escape. Here, we explored the predictive value of the hypoxic model in the immune microenvironment.

**Fig. 4:**
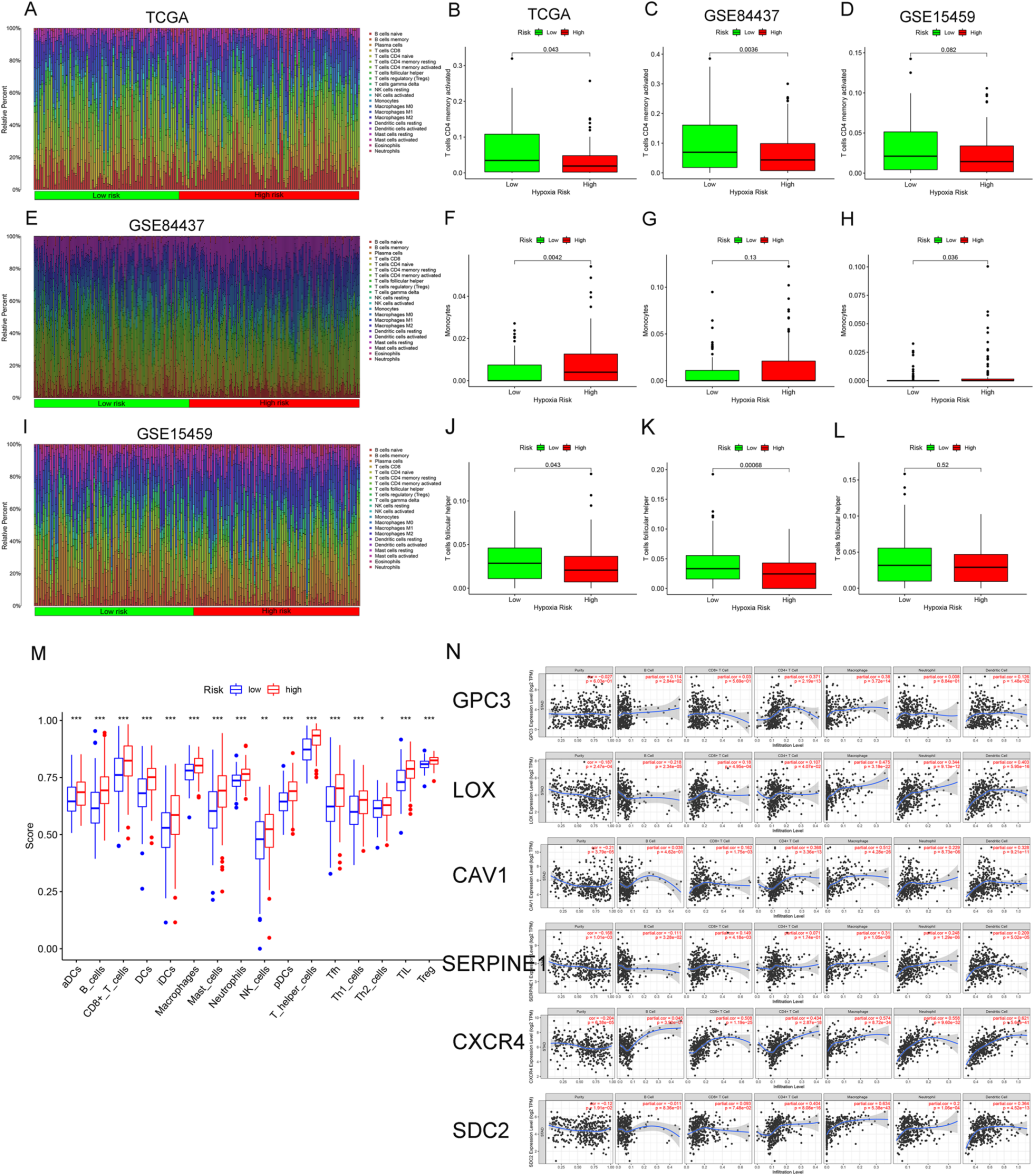
Immune cells in low- and high-hypoxic risk GC patients. **A**, **E** and **I** Proportion of immune infiltration in high- and low-hypoxic risk patients in TCGA and GEO databases. **B**–**D** Box plots visualizing significantly T cells CD4 memory activated between high- and low-hypoxic risk patients in TCGA and GEO databases. **F**–**H** Box plots visualizing monocytes between high- and low-hypoxic risk patients in TCGA and GEO databases. **J**–**L** Box plots visualizing significantly T cells follicular helper between high- and low-hypoxic risk patients in TCGA and GEO databases. **M** Infiltration levels of immune cell types by ssGSEA in TCGA;*P < 0.05, **P < 0.01, ***P < 0.001. **N** Correlation between 6 hypoxia genes and immune cell infiltration in GC patients

Using the CIBERSORT method combined with the LM22 gene matrix, we evaluated the differences in immune infiltration of 22 immune cell types of GC patients in high- and low-risk groups from TCGA. The result of 22 immune cells in the TCGA group was displayed in Fig. 4A. We also depicted the immune cells in the validation sets (Fig. 4E, I). Immune cells in patients at high-hypoxia-risk are suppressed, such as T cells CD4 memory activated and T cells follicular helper (Fig. 4B, J). Besides, the proportion of monocytes in high-hypoxia-risk group was significantly higher (Fig. 4F). Furthermore, same results were found in the two independent validation cohorts in GSE84437 (Fig. 4C, G, K) and GSE15459 (Fig. 4D, H, L). The results of ssGSEA analysis further suggested that the infiltration levels of B cells, macrophages, neutrophils, T helper cells, NK cells and regulatory T cells (Tregs) were significantly increased in the high-hypoxia-risk group (Fig. 4M). We also applied the TIMER tool to analyze the association between hypoxic genes and immune cell infiltration. The six hypoxic genes were positively correlated to CD4+ T cells, CD8+ T cells, macrophages, neutrophils and dendritic cells. In B cells, some genes were positively correlated (GPC3, CAV1, CXCR4), and some were negatively correlated (LOX, SERPINE1, SDC2) (Fig. 4N).

And expression levels of negative regulatory genes in high- and low-risk hypoxic groups were described both in TCGA and GEO cohorts (Additional file 1: Fig. S1A–C). Meanwhile, HER-2 and PD1/PD-L1were found to be associated with hypoxia-risk (Additional file 1: Fig. S1D–I). And violin plots showed differences in TIDE (Additional file 1: Fig. S1J), immune exclusion (Additional file 1: Fig. S1K), and dysfunction (Additional file 1: Fig. S1L) between the two risk groups (*P < 0.05, **P < 0.01, and ***P < 0.001).

### DNA methylation, mutational risk and expression validation

**Fig. 5:**
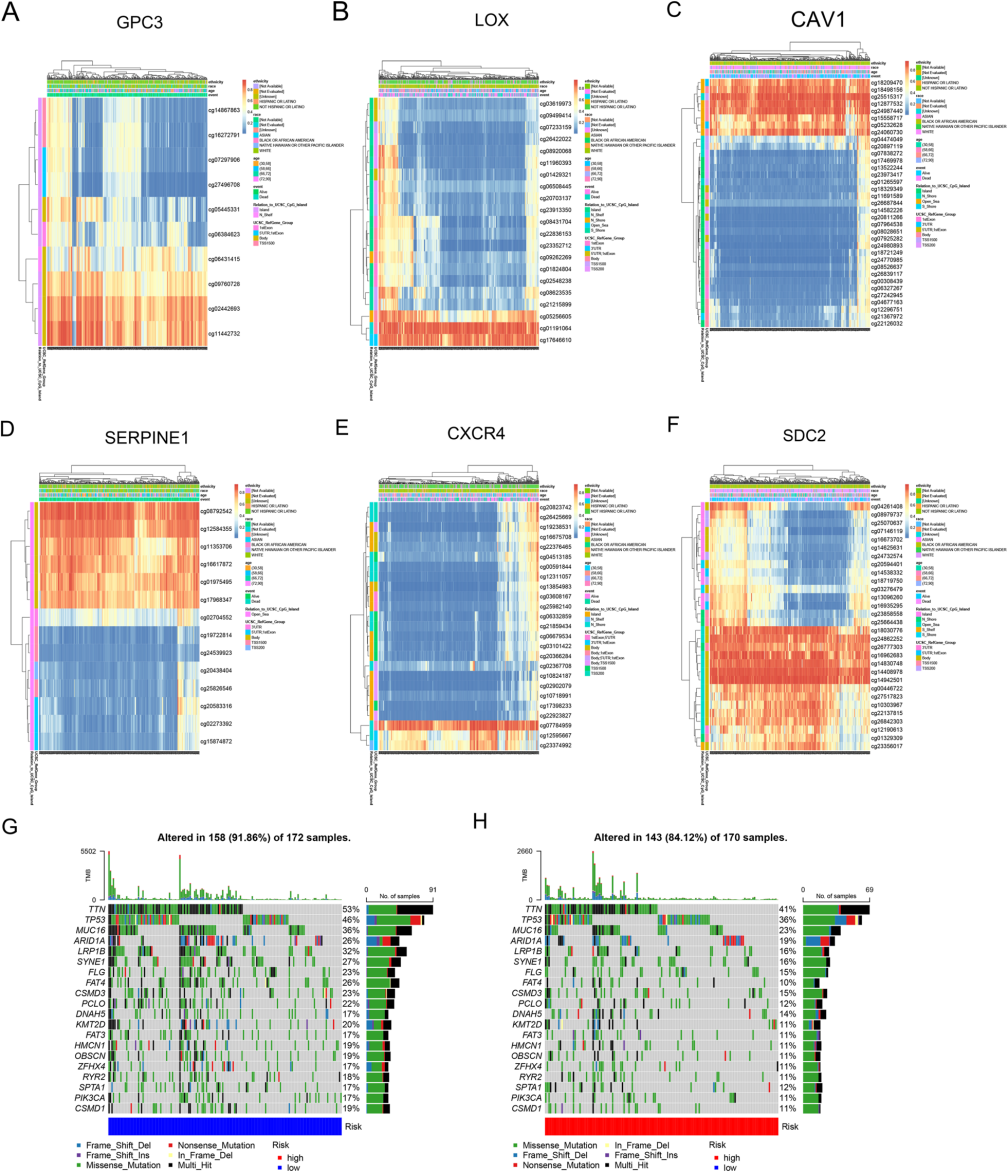
Function and mutational risk of prognostic models. **A**–**F** The heat maps of DNA methylation clustered expression level of GPC3, LOX, CAV1, SERPINE1, CXCR4, SDC2. **G**–**H** Waterfall plots illustrating the mutation status of genes with high mutation rates in the low- and high-risk group

Hypoxic genes are transcribed into proteins after various modifications such as methylation. MethSurv analysis can implicate the prognostic value of DNA methylation in GC. The DNA methylation heatmap showed that cg11442732 of GPC3, cg01191064 of LOX, cg25515317 of CAV1, cg08792542 of SERPINE1, cg07784959 of CXCR4 and cg14942501 of SDC2 had the highest DNA methylation levels in GC (Fig. 5A–F). Overall, we found that 8 CpGs in GPC3, 15 CpGs in LOX, 14 CpGs in CAV1, 13 CpGs in SERPINE1, 12 CpGs in CXCR4 and 23 CpGs in SDC2 were significantly associated with prognosis of GC (Additional file 2: Table S1). Furthermore, we generated two waterfall plots to explore detailed gene mutation risk in high- and low-risk groups (Fig. 5G, H). We found a higher mutation frequency in the low-risk group. And TTN, TP53, and MUC16 were the genes with the highest mutation frequencies in both high- and low-risk groups.

**Fig. 6:**
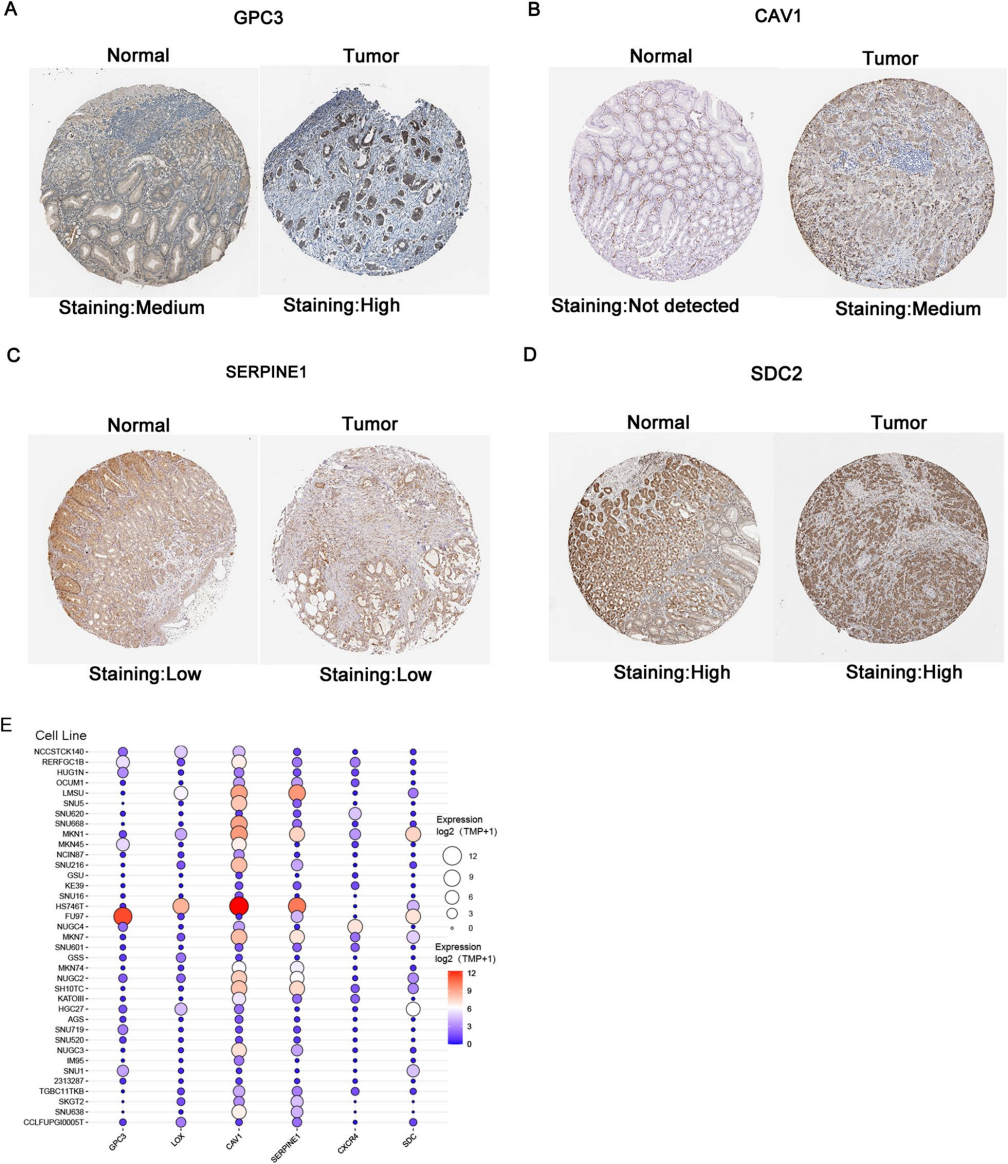
Immunohistochemistry (IHC) results from the Human Protein Atlas (HPA). **A**–**D** Validation of GPC3, CAV1, SERPINE1, SDC2 in turquoise module by The HPA. **E** Expression levels of six hypoxic genes in the Gastric Adenocarcinoma cell line. The X axis represents genes, and the Y axis represents 37 kinds of cell lines. The size and color of the circle indicate the expression level. Red is for high expression, blue is for low

Moreover, the protein levels of immunohistochemistry (IHC) staining obtained from the Human Protein Atlas (HPA) database showed that the expression of the two hypoxic genes (GPC3 and CAV1) were significantly higher in tumor tissues than in normal tissues (Fig. 6A, B), which was consistent with that at the transcriptional level. And two hypoxic genes (SERPINE1 and SDC2) were found with no obvious difference in tumor tissues and normal tissues (Fig. 6C, D). Among the six hypoxic genes, two genes (LOX and CXCR4) were not reported in the HPA database.

In addition, what we found in 37 types of cell lines was as follows: GPC3 was highly expressed in FU97 cell line, and LOX was highly expressed in HS746T cell line. Furthermore, the expression of CAV1 and SERPINE1 was relatively elevated in many GC cell lines (Fig. 6E).

## Discussion

GC is a tumor with poor prognosis. In the past decades, there have been many prognostic models for GC patients [14]. With the deepening of research, it has been found that not only gene mutation exists in cancer, but also the surrounding microenvironment changes [15, 16]. The TME plays an important role in the occurrence, progression and migration of cancer cells. In this study, we found that the hypoxic state of the TME has an important effect on the prognosis of GC patients. Based on the TCGA database, we identified a prognostic model consisted of 6 hypoxia-related genes (GPC3, LOX, CAV1, SERPINE1, CXCR4, SDC2) that could predict OS for GC patients.

The hypoxic microenvironment has an impact on immune cells. The activation of tumor antigen-specific T cells and monocytes is a key incident in anti-tumor immune surveillance [17]. Preceding work has shown that hypoxia could inhibit the growth and activation of T cells [18]. For example, hypoxia induces mitochondrial defects and promotes the exhaustion of T cells in TME through the MYC-regulated pathway [19]. Correspondingly, our research also find that T cells are suppressed in high-risk hypoxia patients. However, the reason for increase of monocytes would need more mechanism studies. Anyhow, it is confirmed that hypoxic microenvironment can regulate the activities of immune cells.

At present, molecular targeted therapy and immunotherapy are catching more and more attention in the treatment of GC [20]. Studies have shown that patients with advanced GC who are HER2 positive can benefit significantly from the combination of anti-HER2 monoclonal antibody therapy with the basic treatment [21]. However, many HER2 positive patients developed drug resistance gradually [22]. Our research suggests that the expression of HER2 is higher in low-hypoxic TME. Thus we hypothesize that resistance of anti-HER2 therapy might be affected by modifying the TME.

Immune checkpoints could promote tumor immunosuppression in the process of canceration [23]. Tumors can escape from immune response by stimulating immune checkpoint targets such as PD1/PD-L1, CTLA-4, LAG-3 and TIM-3 [24–26]. The latest research recommends anti-PD-1 and PD-L1 as the third-line regimen for advanced GC [27]. It is also reported that the hypoxic environment in tumors is associated with the decreased sensitivity of PD-1 signal activation [28]. Moreover, hypoxia can regulate the distribution of PD-L1 at the transcriptional level [29], and giving tissue more oxygen can enhance immunotherapy [30]. Our research found that the expression of PD-1/PD-L1 was increased under hypoxic situation in TCGA database. While the expression of PD-1/PD-L1 was detected with no definitely consistent decrease or increase in high- and low-hypoxic groups in the GEO database. This fully reflects the extreme heterogeneity of GC, and provides evidence of using anti-PD-1/PD-L1 as a third-line treatment option. In other words, we might hypothesize that improving the hypoxic environment could benefit to the immunotherapy in GC. And hypoxia ameliorating agent is hopeful to provide potential value for GC immunotherapy.

However, this study has some limitations. First of all, although two external verifications were conducted in this study, some important clinical information might be missing while collecting characteristic retrospectively from public databases. And it is difficult to cover all of the patients in different geographic regions. Secondly, we did not take into account that the TME status may be various in different tumor regions, such as tumor core and infiltrating edges. Since the samples used for analysis were collected from the core of the tumor, it is impossible to evaluate the immune and hypoxic status of different areas in tumor. In the future, we are looking forward to better-designed multicenter prospective study.

## Conclusion

We establish and validate a hypoxic risk model composed of six hypoxia-related genes. This model can not only assess the prognosis of GC patients, but also evaluate the hypoxia and immune microenvironment in GC. This allows for novel insights for targeted therapy and immunotherapy for GC. We believe that regulating hypoxic microenvironment has a potential ability to enhance the effect of targeted therapy or immunotherapy for GC.

## Supplementary Information

**Additional file 1: Fig. S1.** Association between hypoxic risk score and immunosuppressive microenvironment, targets, immune checkpoints. **A**–**C** Heat map of the gene profiles involved in the negative regulation of Cancer-Immunity Cycle in high- and low-hypoxic risk groups in the TCGA and GEO databases. **D**, **F** and **H** Correlation between HER-2/PD-1/PD-L1 expression and hypoxic risk score. **E**, **G** and **I** HER-2/PD-1/PD-L1 expression levels in high and low hypoxic risk groups. **J**–**L** TIDE, Immune exclusion and Dysfunction in high- and low-hypoxic risk groups. *P < 0.05, **P < 0.01, and ***P < 0.001.

**Additional file 2: Table S1.** The prognostic value of CpG in the hypoxic genes by MethSurv (*P* < 0.05).

## Data Availability

The datasets generated and analyzed during the current study are available in the TCGA repository (https://portal.gdc.cancer.gov) and GEO database (https://www.ncbi.nlm.nih.gov/geo/).
