# Supplementary figures and images for: A signature of six-hypoxia-related genes to evaluate the tumor immune microenvironment and predict prognosis in gastric cancer
Source: BMC Med Genomics. 2022 Dec 16;15:261. doi: 10.1186/s12920-022-01411-9 (PMC9755770; doi:10.1186/s12920-022-01411-9)

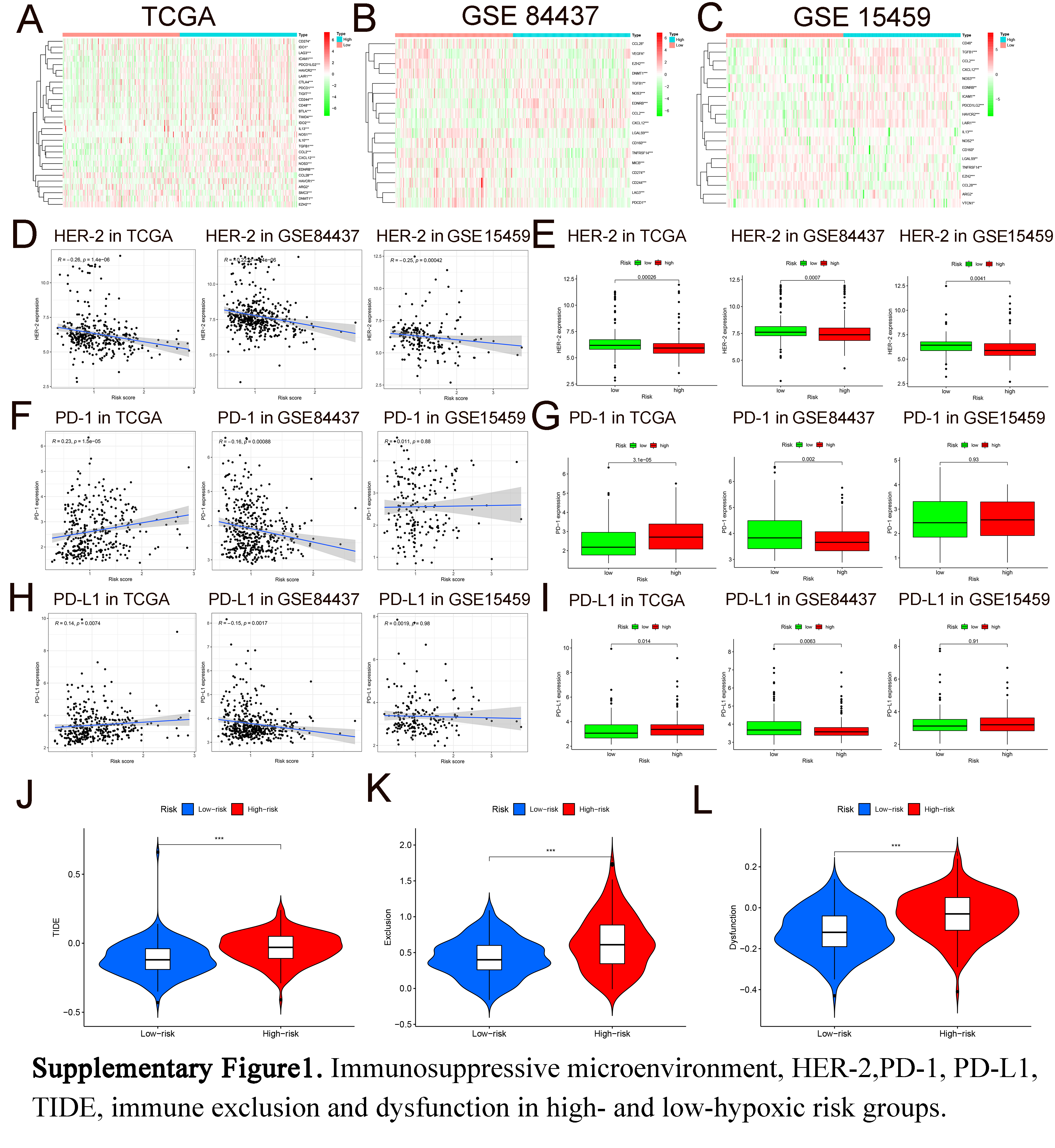

Supplement: Supplementary file 1 — Additional file 1: Fig. S1. Association between hypoxic risk score and immunosuppressive microenvironment, targets, immune checkpoints. A–C Heat map of the gene profiles involved in the negative regulation of Cancer-Immunity Cycle in high- and low-hypoxic risk groups in the TCGA and GEO databases. D, F and H Correlation between HER-2/PD-1/PD-L1 expression and hypoxic risk score. E, G and I HER-2/PD-1/PD-L1 expression levels in high and low hypoxic risk groups. J–L TIDE, Immune exclusion and Dysfunction in high- and low-hypoxic risk groups. *P < 0.05, **P < 0.01, and ***P < 0.001. [file 12920_2022_1411_MOESM1_ESM.tif]
